# Co-existence of a novel plasmid-mediated efflux pump with colistin resistance gene *mcr* in one plasmid confers transferable multidrug resistance in *Klebsiella pneumoniae*

**DOI:** 10.1080/22221751.2020.1768805

**Published:** 2020-05-31

**Authors:** Shijun Sun, Hua Gao, Yudong Liu, Longyang Jin, Ruobing Wang, Xiaojuan Wang, Qi Wang, Yuyao Yin, Yawei Zhang, Hui Wang

**Affiliations:** Department of Clinical Laboratory, Peking University People’s Hospital, Beijing, People’s Republic of China

**Keywords:** Plasmid-mediated tigecycline-resistant *K. pneumoniae*, *tmexCD1-toprJ1* efflux pump, *mcr-8·5*, *tmexCD1*-*mcr* co-harbouring plasmid, CRKP

## Abstract

Tigecycline is considered one of the last-resort antimicrobials for carbapenem-resistant *K. pneumoniae*. Plasmid-mediated tigecycline resistance remains largely unclear. Here, by utilizing whole genome sequencing, we report a plasmid-mediated tigecycline resistance mechanism, a 6,489 bp Resistance-nodulation-division family (RND) efflux pump (*tmexCD1-toprJ1 pump*), that confers transferable tigecycline resistance in *K pneumoniae* isolated from patients and chickens. In addition, we identified high prevalence of the plasmids co-harbouring both *tmexCD1-toprJ1* pump and *mcr* (*tmexCD1-mcr* co-harbouring plasmid) from human in our nationwide collection. Even worse, the *tmexCD1-toprJ1* and *mcr* co-harbouring plasmid was also co-existed with *bla*_NDM_-harbouring IncX3 plasmid in the same host, resulting in pandrug resistance. Phylogenetic analysis suggested that the plasmid-borne *tmexCD1-toprJ1* originated from the chromosome of *Aeromonas* spp. through Tn*5393*-mediating translocation. Both plasmid-harbored *tmexCD1-toprJ1* gene and *mcr-8* likely originated from animal isolates and then spread to human. Our findings highlight a substantial threat of *tmexCD1-toprJ1-mcr8* co-harbouring IncFIA/IncFII plasmid to public health due to their mobile resistance to both tigecycline and colistin, emphasizing an urgent need for further global surveillance on this plasmid.

## Introduction

Carbapenem-resistant *K. pneumoniae* (CRKP), which account for 70–90% of clinical carbapenem-resistant *Enterobacteriaceae* (CRE) infections [1, 2], poses a serious threat to global public health [3, 4]. A number of studies have shown that infections caused by CRKP are associated greater disease severity, more comorbid conditions and increased mortality [5, 6]. Tigecycline and colistin have been regarded as the “last resort” antimicrobials to fight against CRKP [7, 8]. Unfortunately, the high prevalence of CRE has inevitably led to increased use of tigecycline and colistin, accelerating the emergence of tigecycline-resistant and colistin-resistant isolates. Remarkably, tigecycline resistance emerged soon after it was introduced into clinical practice in 2005. Chromosomal mutations, including overexpression of efflux pumps or mutations in the ribosome, have long been regarded as the major mechanisms leading to tigecycline-resistance in *K. pneumoniae* [9]. Emergence and rapid global dissemination of colistin-resistant *K. pneumoniae*, which is due to plasmid-mediated colistin resistance (*mcr*) genes further worsened the situation [10, 11]. In fact, plasmid-mediated resistance to several important antimicrobial classes, such as broad-spectrum beta-lactams and colistin have been found to facilitating resistant gene transfer from animals-derived *Enterobacteriaceae* to human-derived *Enterobacteriaceae* isolates [12]. Of note, recent studies have shown that plasmid-mediated tigecycline resistance genes *tet*(X3), *tet*(X4), *tet*(X5) and *tet*(X6) are predominantly present in *Enterobacteriaceae* and *Acinetobacter* from human and animals [13–20]. Interestingly, until now, only one *tet*(X3)-positive tigecycline-resistant *K. pneumoniae* were identified from a porcine caecum sample, and it remains unclear whether *tet*(X3) is present in human isolates. In the present study, we aimed to further investigate the mechanisms on plasmid-mediated tigecycline resistance by whole genome sequencing. In addition, we investigated whether plasmid-mediated tigecycline resistance co-existed with plasmid-mediated colistin resistance in the same isolate.

## Materials and methods

### Sample collection and bacterial strains

We collected five isolates of *K. pneumoniae* (KA1-KA5) with similar genetic background but substantial differences in resistance to tigecycline and colistin from chickens on the same farm in Shandong Province. Through whole-genome sequencing and bioinformatic analysis, we found the difference in tigecycline resistance was associated with a novel RND family pump, which we initially named as *pmexAB-oprY*. During submission of this manuscript, another group reported the same pump [21]. To avoid any potential confusion in the future studies on this pump, we changed the name of this pump to *tmexCD1*-*toprJ1*, in consistent with Lv et al. [21]. Then, we screened the *tmexCD1-toprJ1* pump in isolates from both chickens and humans from several surveillances. The chicken-derived *K. pneumoniae* were from the animal surveillance from 2013 to 2016, which included a total of 125 *K. pneumoniae* isolated from animals from 4 farms in 3 provinces in China; and the human-derived *K. pneumoniae* were from CMSS (Chinese Meropenem Surveillance Study) and CRE network from 2010 to 2018, which included a total of 3047 *K. pneumoniae* isolated from inpatients from 27 provinces in China. The bacteria identification in this study was performed by MALDI-TOF MS (Bruker Daltonik GmbH, Bremen, Germany).

Patients information were not included in this study; thus, ethical approval was waived (Acceptance number of Ethics Review Committee of Peking University People's Hospital: 2016-PHB-135). All tigecycline-non-susceptible or colistin-non-susceptible isolates, which were determined as tigecycline MICs≥4 μg/ml or colistin MICs≥4 μg/ml, were screened for *tmexCD1-toprJ1* and *mcr-8* by PCR, followed by Sanger sequencing. All of the primers used are listed in the appendix (Table S1). *E coli* J53 (sodium azide resistance) was used as the recipient strain in the conjugation experiment, and *E. coli* DH5α and *K. pneumoniae* ATCC 13883 were used as the recipient strains in the electroporation experiment.

### The antimicrobial susceptibility testing

The MICs were determined by the agar dilution and broth microdilution according to the Clinical and Laboratory Standards Institute (CLSI) guidelines. ATCC 25922 (Escherichia *coli*) and ATCC 27853 (*Pseudomonas aeruginosa*) served as quality control strains for susceptibility testing. The breakpoints of tigecycline and colistin for *Enterobacteriaceae* were interpreted according to the European Committee on Antimicrobial Susceptibility Testing (EUCAST) guidelines and CLSI guidelines, respectively. Tigecycline epidemiological cut-off values (ECOFFs) for *Enterobacteriaceae* were set at >2 μg/ml for Enterobacteriaceae by EUCAST (https://eucast.org/clinical_breakpoints/), while colistin breakpoints for Enterobacteriaceae were set at >4 μg/ml by CLSI.

### Conjugation

The transferability of tigecycline resistance genes was determined by the conjugation assay on the tigecycline resistance strains and azide-resistant *E. coli* J53 by filter mating. The donor and recipient strains were mixed at a ratio of 1:3 on a microporous membrane for 12 h. The transconjugants were then selected on China blue agar plates supplemented with either tigecycline (1 μg/ml) and azide (100 μg/ml), or with colistin (4 μg/ml) and azide (100 μg/ml). The transconjugants were confirmed by MALDI-TOF MS and PCR with specific primers (Table S1) and sequencing. Antimicrobial susceptibility was determined using the broth microdilution method.

### Subcloning experiments

To confirm the role of *tmexCD1-toprJ1* in tigecycline resistance, a 7,205 bp full-length ORF of *tmexCD1-toprJ1* was amplified by PCR (Table S1). Subsequently, 0·3 pmol of purified PCR product was incubated with 1 μL T-Vector pMD-19 (TaKaRa) and 5 μL DNA Ligation Mighty Mix (TaKaRa) at 16°C overnight. The recombinant plasmid pUC19-*tmexCD1-toprJ1* was then transformed into competent *E. coli* DH5α by incubating on ice for 30 min followed by incubating for 45 s at 42°C. Immediately thereafter, the DH5α was transferred to SOC medium (TaKaRa) at 37°C for one hour and then was coated on Luria–Bertani Agar (LB) medium containing 100 μg/ml of ampicillin at 37°C overnight. Visible strains after overnight were verified by PCR and then subjected to susceptibility testing using broth microdilution method. The recombinant vector was then extracted and transferred into *K. pneumoniae* ATCC 13883 via electroporation.

### ***Acrb*** and ***ramA*** gene expression analysis using real-time reverse transcription PCR (RT–PCR)

The expression levels of *acrAB* efflux pump genes were analysed by RT–PCR. The primers for *acrB*, *ramA* and the *rrsE* were used as previously described [22]. Briefly, bacteria were grown aerobically in LB broth until mid-log phase. DNase-treated RNA templates were prepared using the RNeasy Kit (Qiagen, Hilden, Germany). cDNA was generated from total RNA using random primer hexamers. RT–PCR was performed using a Roche 480 thermocycler with a SYBR green PCR master mix (TaKaRa). The PCR programme consisted of 5s at 95°C, followed by 40 cycles of 15 s at 95°C and 31 s at 58°C. Each sample was run in triplicate.

### Growth rate assay

Bacteria were inoculated into 5 mL of Mueller-Hinton broth and incubated overnight at 37°C. Overnight cultures of *E. coli* J53 and the transconjugants were diluted 1:10000 in Mueller-Hinton broth, and growth curves were performed in triplicate by incubating the cultures for 24 h at 37°C. Bacterial growth was monitored by measuring the optical density of the culture at 620 nm by Varioskan Flash (Thermo Fisher Scientific^TM^, Shanghai, China).

### Whole-genome sequencing

Total genomic DNA were extracted using the TIANamp Bacteria DNA Kit DP302 (Tiangen Biotech, Beijing, China) followed by genomic DNA sequencing with Illumina HiSeq X Ten platform, which produced 150-bp paired-end reads and at least 100-fold coverage of raw reads. 20 of the isolates were also extracted by the QIAGEN Large-Construct kit (Qiagen Sciences, Germantown, MD, USA) and sequenced by the PacBio RS II system (Pacific Biosciences) with a 10-kb size library and P6/C4 chemistry. The hybrid assembling was performed using Unicycler v0·4·6. All draft genome sequences were deposited into NCBI Genome database, and organized under BioProject ID PRJNA595047. All resistance genes were detected by the Resfinder (https://cge.cbs.dtu.dk/services/ResFinder/) and Basic Local Alignment Search Tool (BLAST).

### Public genomic database searching

The *tmexCD1-toprJ1* efflux pump and *mcr-8* sequences were queried against NCBI RefSeq Assembly database. The meta information including collection date, organism, source and et al of all subject strains was collected. Any strains without year of collection were discarded.

### Sequence annotation

The genome sequence was annotated by Rapid Annotation Subsequencing Technology (RAST) (http://rast.nmpdr.org/), Prokka v1·12 and BLAST, when applicable. Comparative analysis was performed by BLAST Ring Image Generator (BRIG), and genetic structures of representative plasmids were generated with DNAPlotter and Vector NTI. For Prokka, the assembly was first annotated by Prokka v1·12, and then combined with the best matches from blast hits (minimal identity 90%, minimal coverage 98%) against several specific databases, including SerotypeFinder, PlasmidFinder, ResFinder, CARD, VirulenceFinder, VFDB, ICEberg and ISFinder. Gene organizations were illustrated using R software.

### Phylogenetic analysis and dating

Longest homology regions containing *tmexCD1-toprJ1* or *mcr-8* were determined based on pairwise Blast. Multiple sequence alignment of the homology regions was constructed by progressiveMauve, and all gaps were removed. Possible recombination events were detected by ClonalFrameML and removed. Phylogenetic tree was then constructed using BEAST under different site models, clock models and tree priors. Best model was selected based on BIC criteria. Divergence dating was inferred from MCMC trees. Phylogenetic tree was illustrated using R ggtree package.

### Role of the funding source

The funder of the study had no role in study design, data collection, data analysis, data interpretation, or writing of the report. The corresponding author had full access to all the data in the study and had final responsibility for the decision to submit for publication.

## Results

### A novel efflux pump gene confers transferable resistance to tigecycline on plasmids

The five *K. pneumoniae* isolates (isolates KA1-KA5, Table 1) from the same farm in Shandong displayed the same multilocus sequence typing (MLST) typing (ST37) but different levels of resistance to tigecycline. We screened major known chromosomal-mediated tigecycline resistance mechanisms, including *ramA*, *ramR* genes and *acrAB* efflux pump, but failed to find any differences between the resistant strains and susceptible strains. We then subjected all the five strains to whole genome sequencing including PacBio Sequencing. Cross-comparison of sequencing data among the five isolates revealed that the chromosome genomes of all isolates were highly similar (degree of matching over 99%) with slight difference in the plasmids. Further analysis of the plasmid sequences between tigecycline-non-susceptible *K. pneumoniae* (TNSKP) and tigecycline-susceptible *K. pneumoniae* (TSKP) from the five isolates motioned above highlighted a 6489 bp resistance-nodulation-cell division (RND) family efflux pump, which we suspected to be related to tigecycline resistance. The novel RND family efflux pump, which consists four ORFs (558, 1164, 3315, and 1434 bp in lengths, respectively), displayed the highest similarity to the *mexCD* efflux pump *nfxB-mexC-mexD-oprJ* from the *mex* family members with consistencies of 54%, 60%, 77% and 67%, respectively. We initially named this pump as *pmexAB-oprY*, which was changed to *tmexCD1*-*toprJ1* in consistent with Lv et al [21].

**Table 1.** Phenotype and genotype of 24 ***Klebsiella pneumoniae*** isolates that carrying ***tmexCD1-toprJ1*** efflux pump or ***mcr-8*** gene in this study.

Isolates No.	Year	Region	Origin	Specimen type	Sequence type	MIC (µg/mL)			resistance gene/plasmid type/plasmid size (bp)		
						TGC	COL	MEM	*tmexCD1-toprJ1*	*mcr*	Carbapenemases genes
KA1	2013	Shandong	Dead chicken	Anal swab	ST37	16	8	0·125	IncFIB(K)/ ∼136095	*mcr-8·1/* IncFIA(HI1)/∼81030	-
KA2	2013	Shandong	Healthy chicken	Anal swab	ST37	1	8	0·125	-	*mcr-8·1/* IncFIA(HI1)/ 95191	-
KA3	2013	Shandong	Dead chicken	Anal swab	ST37	8	4	0·064	IncFIB(K)/ 189110	*mcr-8·1/* IncFIA(HI1)/ 72972	-
KA4	2013	Shandong	Chicken	Manure	ST37	8	2	0·125	IncFII(K)/ 175838	*-*	-
KA5	2013	Shandong	Healthy chicken	Anal swab	ST37	1	4	0·064	*-*	*mcr-8·1*/ IncFIA(HI1)/ 95191	-
KA6*	2016	Shandong	Chicken	Manure	ST1	16	16	0·032	IncFIA/134000	*mcr-8·1/*IncFIA/134000	-
KA7*	2016	Shandong	Chicken	Manure	ST11	32	32	0·032	IncFIA/112696	*mcr-8·1/*IncFIA/112696	-
KA9*	2016	Shandong	Chicken	Manure	ST37	16	64	8	IncFII(K)/ 120427	*mcr-8·5*/IncFII(K)/ 120427	*bla*_NDM-5_*/* IncX3/48100
KA10*	2016	Shandong	Chicken	Manure	ST37	16	32	8	IncFII(K)/ 132165	*mcr-8·5*/IncFII(K)/ 132165	*bla*_NDM-5_*/* IncX3/54737
KA11	2016	Shandong	Chicken	Manure	ST726	8	256	8	IncFIA(HI1)/ 169808	*mcr-3·11*/IncHI1B/70703	*bla*_NDM-5_*/* IncX3/46161
KA12	2016	Shandong	Chicken	Manure	ST726	8	256	8	IncFIA(HI1)/ 169593	*-*	*bla*_NDM-5_*/* IncX3/46161
KA13	2016	Henan	Chicken farm	Drinking water	ST726	4	256	0·032	IncFIA/169808	*-*	-
KA14	2016	Shandong	Chicken	Manure	NT	1	4	0·032	-	*mcr-8·1/* IncFIA(HI1)/ ∼78204	-
KH3	2014	Beijing	Inpatient	Urine	ST37	8	16	4	IncFIB(K)/ 199562	*mcr-8·1*/IncFIA(HI1)/65808	*bla*_NDM-1_*/* IncA/C2/170776
KA15	2013	Shandong	Dead chicken	Anal swab	ST2018	0·5	32	0·125	-	*mcr-8·1*/IncFIA(HI1)/∼114644	-
KA16	2013	Shandong	Dead chicken	Anal swab	ST2018	0·5	32	0·064	-	*mcr-8·1*/ IncFIA(HI1)/ 116873	-
KA17	2013	Shandong	Healthy chicken	Anal swab	ST2018	1	32	4	-	*mcr-8·1*/ IncFIA(HI1)/ 114963	-
KA18	2013	Shandong	Healthy chicken	Anal swab	ST2018	0·5	32	4	-	*mcr-8·1*/ IncFIA(HI1)/ 116873	-
KA19	2013	Shandong	Healthy chicken	Anal swab	ST2018	1	32	0·064	-	*mcr-8·1*/ IncFIA(HI1)/ 116873	-
KA20	2013	Shandong	Breeding chicken	Anal swab	ST2018	0·5	32	0·125	-	*mcr-8·1*/ IncFIA(HI1) ∼114471	-
KH4	2014	Beijing	Inpatient	Urine	ST11	2	16	8	-	*mcr-8·1/*IncFIA/122389	*bla*_NDM-1_*/* IncA/C2/165250
KH5	2014	Henan	Inpatient	Urine	ST37	4	16	4	-	*mcr-8·1*/ IncFII(K)/ ∼106676	-
KH6	2015	Shandong	Inpatient	Sputum	ST395	2	8	8	-	*mcr-8·1*/IncFII(K)/ ∼99252	-
KH7	2017	Neimengu	Inpatient	Urine	ST423	1	8	0·032	-	*mcr-8·2*/ IncFIA(HI1)/ ∼94091	-

Antimicrobial agents are abbreviated as follows: TGC*,* tigecycline; COL*,* colistin; MEM, meropenem

**NT**: novel type.

* means *tmexCD1-toprJ1* efflux pump and *mcr-8* gene of the isolate are on the same plasmid.

**∼** refers to the plasmid size estimated from the Illumina sequencing data.

### The prevalence of ***tmexCD1-toprJ1*** in TNSKPs

The sequencing data also revealed that four of 5 isolates (KA1, KA2, KA3 and KA5) contained *mcr-8·1*-harbouring plasmids, which led those isolates resistant to colistin. Most importantly, 2 isolates (KA1 and KA3) contained both *mcr-8·1* and *tmexCD1-toprJ1*. We then did a retrospective screening on a large collection of isolates from our routine surveillance networks, including animal Network, CMSS and CRE Network. A total of 257 TNSKPs were obtained from 10536 Gram-negative bacilli, including 21 TNSKPs from animals and 236 TNSKPs from patients with various infections. Additional 8 isolates from chickens and 6 isolates from inpatients among the 257 TNSKPs were identified positive for the *tmexCD1-toprJ1* gene. The prevalence of *tmexCD1-toprJ1* in TNSKPs were 52·4% in animals and 2·5% in patients, respectively (table S3). Notably, all of *tmexCD1-toprJ1*-positive isolates were resistant to tigecycline, further reinforcing a link between *tmexCD1-toprJ1* and tigecycline resistance.

### Discovery of a new ***mcr-8*** variant and the co-existing of multiple drug resistance genes in same isolates

**Figure 1.**
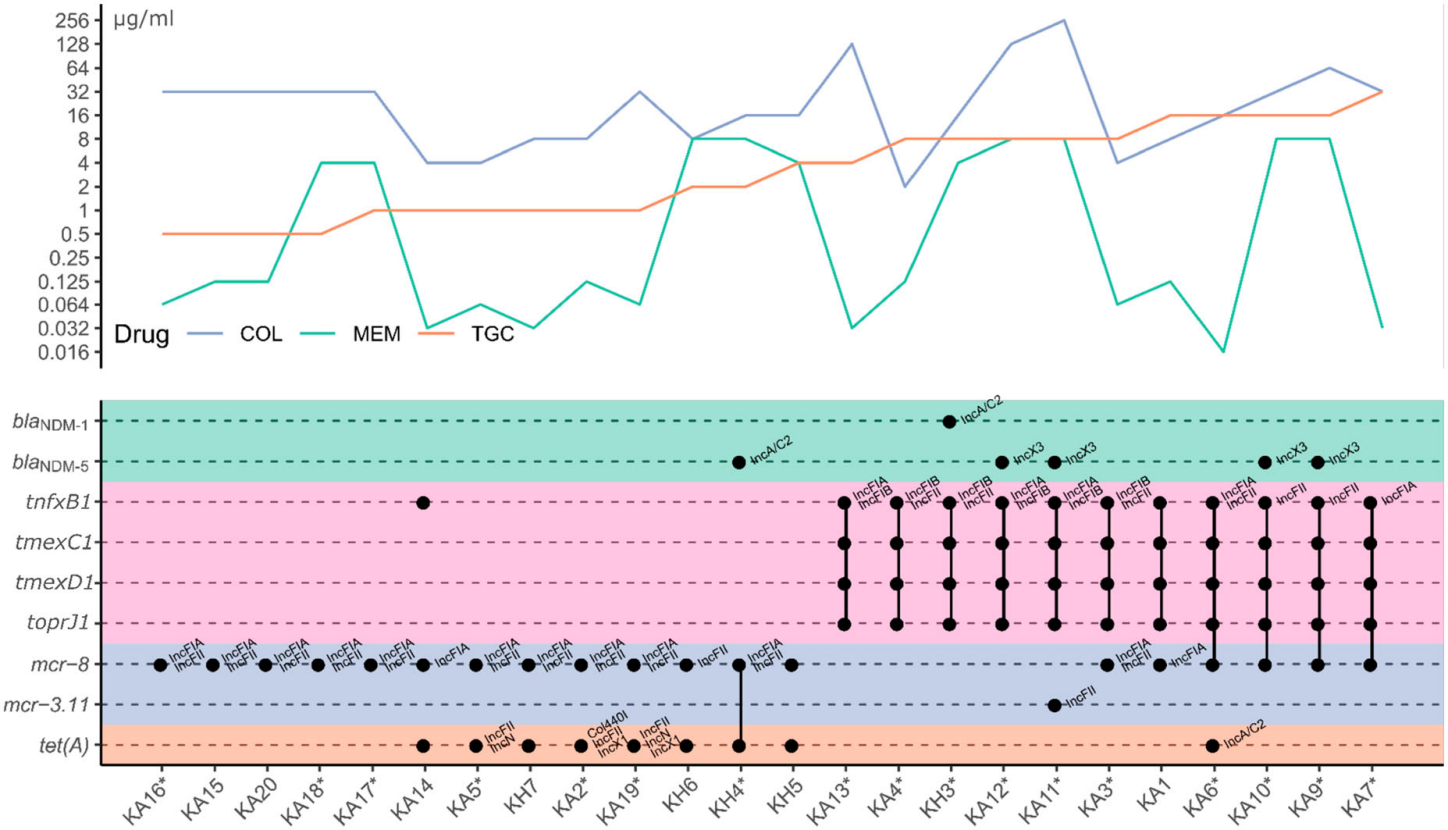
Genetic features of AMR genes and resistance phenotype. Upper panel is the MIC curves for different drugs. Lower panel is the genetic features of related AMR genes. Each dot represents the existence of the gene, and the known replication origin type is labelled at top-right. Linked dots by vertical line mean they are co-located on the same plasmid. The star after sample name mark the availability of PacBio sequencing.

Importantly, we noticed that among the 17 *tmexCD1-toprJ1*-positive isolates (14 isolates from the retrospective screening mentioned above and 3 isolates from the first 5 isolates that were positive for *tmexCD1-toprJ1*), 7 isolates (41·2%, 7/17) were positive for both *mcr-8 (mcr-8·1,* and *mcr-8·5)* and *tmexCD1-toprJ1.* In addition to *mcr-8, mcr-3.11* was identified in one isolate (KA11)*. mcr-8·5* (GenBank accession number MN836537), a new *mcr-8* variant found in this study, exhibited 6 single nucleotide mutation sites (C152T, G694T, T1462A, A1559T, C1578T, A1690G) compared to *mcr-8·1*, leading to changes in 5 amino acids (A51V, A232S, F488I, Y520F, N564D). The high prevalence (41·2%) of *tmexCD1-toprJ1-mcr8*-double positive isolates greatly attracted our attention. To understand the relationship between *tmexCD1-toprJ1* and *mcr-8* in the same strain, we selected 19 isolates (11 isolates from the 17 *tmexCD1-toprJ1*-positive isolates mentioned above and 8 isolates were *mcr*-positive but *tmexCD1-toprJ1*-negative strains with similar background with *tmexCD1-toprJ1*-positive isolates from our surveillance network), which were positive for *tmexCD1-toprJ1* and/or *mcr-8*, for whole genome sequencing to investigate the underlying mechanisms of tigecycline resistance (Figure 1). As shown in Figure 1, the presence of *tmexCD1-toprJ1* genes is associated with higher levels of tigecycline MICs, but not with colistin MICs or meropenem MICs. We also compared the expression levels of chromosomal-derived tigecycline resistance mechanism, and failed to detect any significant differences between *tmexCD1-toprJ1*-positive TNSKPs and TSKP, while *tmexCD1-toprJ1*-negative tigecycline-resistant *K. pneumoniae* displayed significantly higher expression levels of *acrB* and *ramA* compared to *tmexCD1-toprJ1*-positive *tigecycline-resistant K. pneumoniae* and TSKP (figure S1), suggesting that tigecycline resistance in *tmexCD1-toprJ1*-positive *tigecycline-resistant K. pneumoniae*s were not due to higher expression of those chromosomal-derived efflux pumps.

Even worse, we found that 40% (4/10) of the strains carrying both *tmexCD1-toprJ1* and *mcr* were also positive for *bla*_NDM_ (Table 1). Of particular clinical relevance is KH3, an isolate from urine sample from an inpatient in Beijing. KH3 was positive for *tmexCD1-toprJ1, mcr-8·1* and *bla*_NDM-1_, making this strain resistant to all clinically available antibiotics (Table 1 and table S4).

### One plasmid carries both *mcr* and *tmexCD1-toprJ1* in 4 isolates from chickens

**Figure 2.**
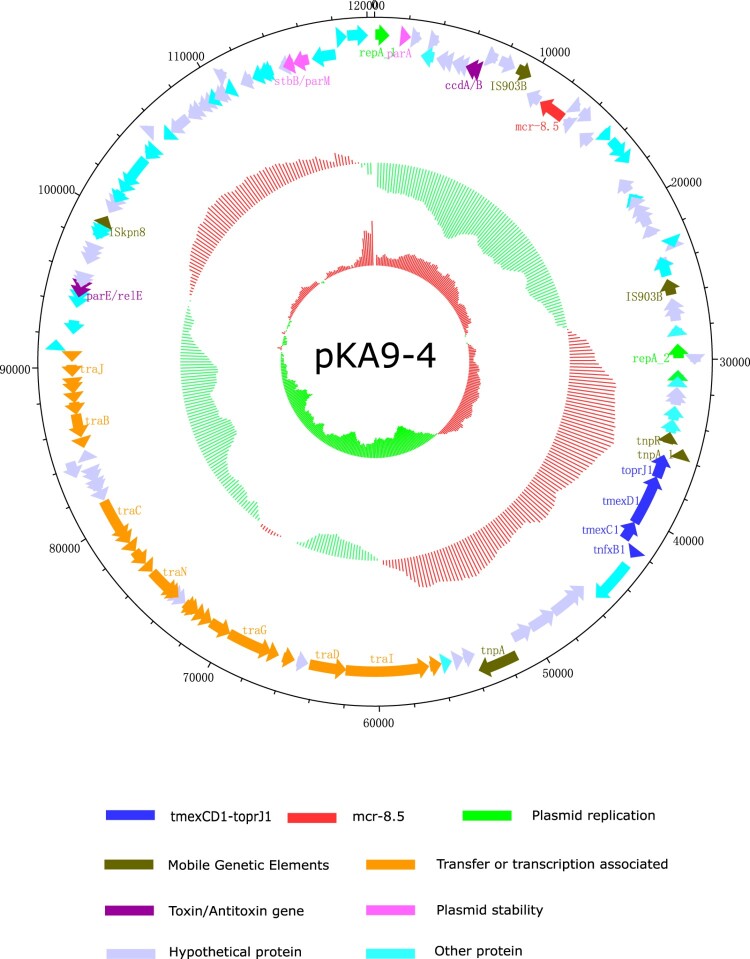
The genetic contents of plasmid pKA9-4. The positions of predicted coding sequences transcribed in a anticlockwise orientation (GenBank accession number: MN832595).

**Figure 3.**
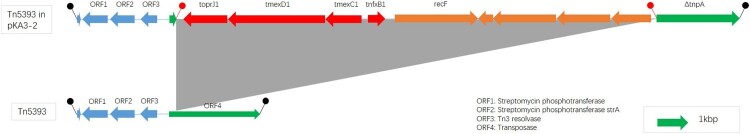
The genetic environment of the *tmexCD1-toprJ1* efflux pump and the new variant of Tn*5393* in pKA3-2. Black lollipop represents the direct repeat (DR) sequence (AACTA) of Tn*5393*, and red lollipop represents the DR sequence (ATCGA) generated when *tmexCD1-toprJ1* is inserted.

Remarkably, further comparative analysis of PacBio and HiSeq sequencing data revealed co-existence of *mcr-8 and tmexCD1-toprJ1* in the same plasmid in four isolates (KA6, KA7, KA9 and KA10). Specifically, *mcr-8·1* co-existed with *tmexCD1-toprJ1* on IncFIA plasmid in two isolates (KA7 and KA9), while *mcr-8·5* co-existed with *tmexCD1-toprJ1* on IncFII(K) plasmid in the other two isolates (KA6 and KA10) (Table 1). For *tmexCD1-toprJ1*-harbouring plasmid, IncFIA was the most popular type, accounting for 45·5% (5/11) of *tmexCD1-toprJ1*-positive isolates, followed by IncFII (27·3%, 3/11) and IncFIB (27·3%, 3/11). Interestingly, all *mcr-8·1* was located in IncFIA, while *mcr-8·5* was located in IncFII(K) plasmid (Table 1). pKA9-4 (GenBank accession number MN832595), a 120-kb IncFII(K) plasmid, displayed 74% query coverage and 97·02% identity to the *mcr-8*-harbouring plasmid pKP91 from *K. pneumoniae* (Genbank accession number, MG736312) by Blast in the NCBI database (Figure 2). In addition, further analysis showed that all the *tmexCD1-toprJ1* efflux pumps were embedded in transposon Tn*5393* (Figure 3). Taken together, these findings demonstrated that the high prevalence of *tmexCD1-toprJ1-mcr8*-double positive *K. pneumoniae* is partially due to dissemination of plasmids that carry both *mcr-8* and *tmexCD1-toprJ1*.

### Function of ***tmexCD1-toprJ1*** and the transferability of the plasmid carrying the ***tmexCD1-toprJ1***

To directly confirm the function of the *tmexCD1-toprJ1* pump, we performed a series of conjugation assays on KA1, KA3 and other three *K. pneumoniae* isolates (KA6, KA7 and KA9), which contained both *mcr-8·1* and *tmexCD1-toprJ1*. Although the conjugation assays on KA1 and KA3 failed, the conjugation assay on other three isolates succeeded. As expected, the plasmid carrying the *tmexCD1-toprJ1* pump greatly increased tigecycline resistance in *E· coli* J53 by at least 8-fold with transfer frequencies of 1·20±0·326 × 10^−7^. We next ligated the *tmexCD1-toprJ1* gene to vector pUC-19 and transferred the plasmid pUC-19*-tmexCD1-toprJ1* into *E· coli* DH5α and *K. pneumoniae* ATCC 13883. The antimicrobial susceptibility results showed that pUC-19*-tmexCD1-toprJ1* increased the tigecycline MIC in DH5α by 8-fold and in *K. pneumoniae* ATCC 13883 by 16-fold, respectively (Table 2). In addition, the reduced susceptibility was also observed for tetracycline and ciprofloxacin (Table 2 and table S2). Taken together, these results show that *tmexCD1-toprJ1* confers mobile tigecycline resistance in the host. Further, the effect of acquiring *tmexCD1-toprJ1*-and/or *mcr-8-*harbouring plasmids on biological fitness cost was evaluated. No significant differences in the growth rates were observed among the recipient strain *J53*, the *mcr-8-*plasmid-transconjugants strain KH3-T and the *tmexCD1-mcr-8*-co-harbouring plasmid plasmid-transconjugants KA6-T (Figure S2). These results indicate that acquiring plasmids co-carrying *tmexCD1-toprJ1* and *mcr-8* does not have a significant effect on fitness cost.

**Table 2.** Antimicrobial susceptibility profiles of the isolates carrying the ***tmexCD1-toprJ1*** and their transconjugants and transformants.

Bacteria	Description	Resistance genes associated with tigecycline, colistin and carbapenem antibiotics	MIC (μg/mL)			
			TGC	COL	MEM	CIP
KA6	Donor of pKA6-2	*tmexCD1-toprJ1 /mcr-8·1*	16	16	0·032	>64
KA6-T	Transconjugants of pKA6-2	*tmexCD1-toprJ1 /mcr-8·1*	2	8	0·032	1
KA7	Donor of pKA7-1	*tmexCD1-toprJ1 /mcr-8·1*	32	32	0·032	>64
KA7-T	Transconjugants of pKA7-1	*tmexCD1-toprJ1 /mcr-8·1*	2	4	0·032	0·5
KA9	Donor of pKA9-4 and pKA9-6-*bla*_NDM_	*tmexCD1-toprJ1 /mcr-8·5 /bla*_NDM_	16	64	8	>64
KA9-T	Transconjugants of pKA9-4 and pKA9-6-*bla*_NDM_	*tmexCD1-toprJ1 /mcr-8·5 /bla*_NDM_	2	4	8	8
KH3	Donor of pKH3-5	*tmexCD1-toprJ1 /mcr-8·1 /bla*_NDM_	8	8	8	>64
KH3-T	Transconjugants of pKH3-5	*mcr-8·1*	0·25	2	0·032	<=0·032
*E.coli* J53	Recipient for conjugation	–	0·25	0·5	0·032	≤0·032
*E.coli* DH5α	Recipient for transformantion	–	0·125	0·5	≤0·016	≤0·032
DH5α+pUC19	Transformants	–	0·125	0·5	≤0·016	≤0·032
DH5α+pUC19-*tmexCD1*	Transformants	*tmexCD1-toprJ1*	1	0·5	≤0·016	0·064
*K. pneumoniae* ATCC13883	Recipient for transformantion	–	0·125	0·25	≤0·016	≤0·032
ATCC13883+pUC19	Transformants	–	0·125	0·25	≤0·016	≤0·032
ATCC13883+pUC19-*tmexCD1*	Transformants	*tmexCD1-toprJ1*	2	0·25	≤0·016	0·064

Antimicrobial agents are abbreviated as follows: TGC, tigecycline; COL, colistin; MEM, meropenem; CIP, ciprofloxacin

### Origin and evolution of ***tmexCD1-toprJ1*** and ***mcr-8***

**Figure 4.**
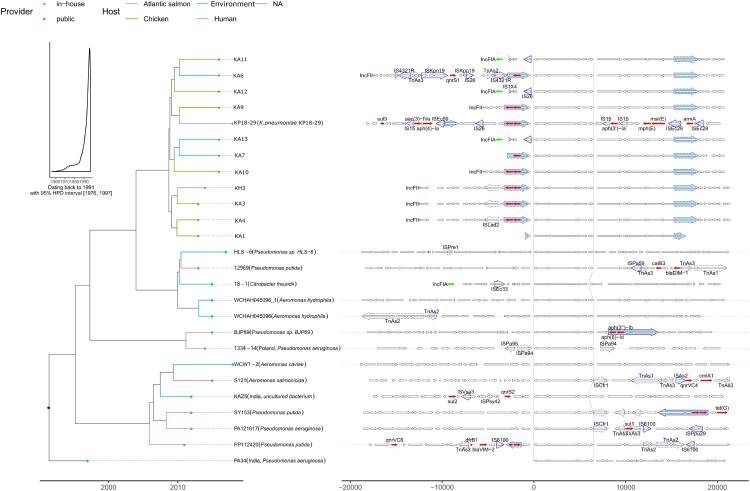
Phylogenetic tree and sequence context comparison of *tmexCD1-toprJ1*. Left panel is the phylogenetic tree inferred by BEAST2. The fill colours of tip nodes represent the sample source, and the line colours of tip branches demonstrate the hosts of strains. The insert histogram is the distribution of dating year for the root node. Right panel shows the sequence context. Thin arrows are genes, and fat arrows are insertion sequences where the dashed lines marks truncated ones. The highlighted (blue filled) insertion sequences are Tn*5393*.

To gain insight into the spatiotemporal origin of the *tmexCD1-toprJ1* gene, NCBI genomic database search was performed for strains containing the *tmexCD1-toprJ1* gene. A total of 15 strains were found based on the criteria that the uploaded sequence from each strain was greater than 20 kb and had 100% coverage of *tmexCD1-toprJ1* gene. Of note, phylogenetic tree and dating were performed based on the *tmexCD1-toprJ1* sequences from 11 TNSKPs in this study and the 15 strains from the NCBI (Figure 4). Interestingly, the *tmexCD1-toprJ1* gene was located on the chromosome in almost all non-*K. pneumoniae* organisms. In contrast, the *tmexCD1-toprJ1* gene was found inside Tn*5393* on plasmid in all *K. pneumoniae* (Figure 4). Importantly, the sequence contexts from *Aeromonas* (WCHAH045096 and WCW1-2) were found more similar to those seen in *K. pneumoniae* than *Pseudomonas* (Figure 4). Thus, it is likely that the plasmid-harbored *tmexCD1-toprJ1* gene originated from the chromosome of *Aeromonas* spp. through Tn*5393* mediated translocation. Of note, there were at least one type of IncFIA plasmid and two types of IncFII plasmids harbouring Tn*5393*::*tmexCD1-toprJ1* (*tmexCD1-toprJ1* inserted in Tn*5393*), indicating this transposon may still possess the translocation capacity. The transposon in IncFIA plasmids (KA11, KA12 and KA13) were truncated from first three ORFs by IS*1X4* and IS*26*, while the transposon in one IncFII plasmid (KP18-29) lost the transposase gene due to the replacement of tandem of insertion sequences. In addition, two strains (KP18-29 and KH3) isolated from human were found being surrounded by chicken-derived strains in the phylogenetic tree, suggesting a possibility of chicken to human transmission, although we cannot exclude the possibility of human to chicken transmission.

**Figure 5.**
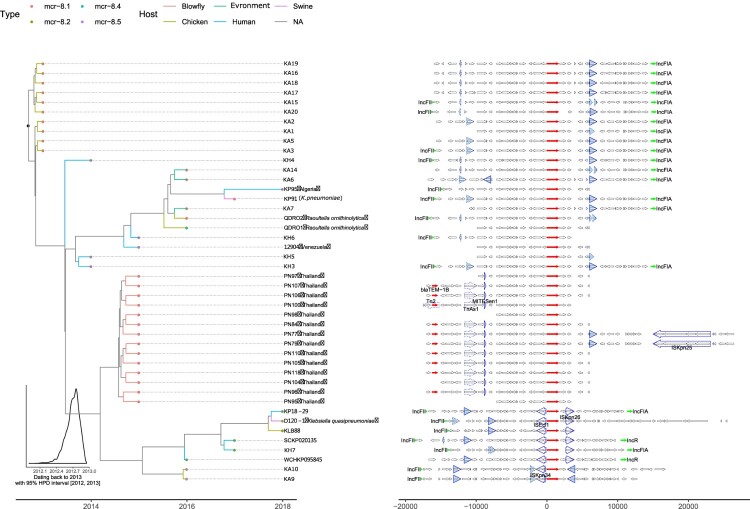
Phylogenetic tree and sequence context comparison of *mcr-8*. Left panel is the phylogenetic tree inferred by BEAST2. The fill colours of tip nodes represent different subtypes of mcr-8, and the line colours of tip branches demonstrate the hosts of strains. The insert histogram is the distribution of dating year for the root node. Right panel shows the sequence context. Thin arrows are genes, and fat arrows are insertion sequences where the dashed lines marks truncated ones. The highlighted (blue filled) insertion sequences are IS*903B*.

The spatiotemporal origin of a new *mcr-8* variant *mcr-8·5* was assessed. Phylogenetic analysis and gene context comparison revealed that *mcr-8·5* clustered with *mcr-8·2* and they shared larger amount of sequence context. Thus *mcr-8·5* was more likely derived from *mcr-8·2*. It is noticeable that, in comparison to mcr-8.1, *mcr-8·2* and *mcr-8·5* were surrounded by complete insertion sequences (IS*Kpn26*, IS*Ecl1* and IS*903B*), which makes them to have higher risk for transposition and dissemination. In addition, we dated the emergence of *mcr-8* back to 2013 (Figure 5). Of note, as the chicken-derived strains were found quite close to that origin time, it is likely that *mcr-8* was originated from animal and subsequently spread to human.

## Discussion

Conjugative plasmids play an essential role in the dissemination of antimicrobial resistance among clinically important pathogens, such as *K. pneumoniae*. In the present study, we identified a plasmid-mediated tigecycline resistance mechanism, mediating by the *tmexCD1-toprJ1* pump, in TNSKPs isolated from both patients and chickens. We also found the presence of plasmids co-harbouring *tmexCD1-toprJ1* and *mcr-8.5* genes*,* which allows simultaneous transmission of tigecycline resistance and colistin resistance in a single transfer event. Given previous experience on the rapid dissemination of carbapenem-resistant plasmids (*bla*_KPC-2_ or *bla*_NDM-1_ plasmids) and colistin-resistant plasmid (*mcr* plasmids), we highly suspect that the emergence of transmissible tigecycline-resistant plasmids (*tmexCD1-toprJ1* plasmids), especially the emergence of plasmid co-harbouring both *tmexCD1-toprJ1* and *mcr*, will largely promote the progression of global pan-drug resistance.

The novel plasmid-harbouring *tmexCD1-toprJ1* pump belonged to a *mex* family pumps. The *mex* family proteins were first reported in the chromosomal genome of Pseudomonas *aeruginosa* as efflux pumps to confers *P. aeruginosa* intrinsic resistance to tetracycline, chloramphenicol and norfloxacin [23]. Interestingly, although the *mex* family genes have been extensive studies in *P. aeruginosa*, in terms of plasmid-harbored *tmexCD1-toprJ1* gene*,* it is more likely originated from the chromosome of *Aeromonas* spp. as assessed by the sequence contexts.

The recent emergence of plasmid-mediated tigecycline resistance genes *tet*(X3) and *tet*(X4) have attracted intense attention [16]. Interestingly, until now, *tet*(X3) and *tet*(X4) were predominantly found in *E. coli*, and have never been reported in tigecycline-resistant *K. pneumoniae* isolated from human origin. In fact, we have screened a total of 161 TNSKPs from patients with various infections and failed to detect any *tet*(X3)/*tet*(X4)-positive TNSKPs (data not shown). In contrast, we found that plasmid-harbored *tmexCD1-toprJ1* gene was present in TNSKP from human origin with a prevalence of 2·5%. In search of the public database, we found a human-derived *K. pneumoniae* plasmid contig containing the same sequence as *tmexCD1-toprJ1* from Henan province (China) in 2018, which has been recently submitted to the NCBI (GenBank accession number MK262712·1) but was not annotated. Although no detailed information is available, the data confirms the presence of *tmexCD1-toprJ1* in *K. pneumoniae* from human origin. Just recently, Lv et al. also found this pump in isolates from human [21]. In our study, *tmexCD1-toprJ1* was first identified in TNSKPs from inpatients in 2012 from two Provinces in China (Jiangsu and Tianjin), and then was found in TNSKP from an inpatient in Beijing in 2014. Of interest, *tmexCD1-toprJ1* was re-emerged in 2018 from an inpatient in Xinjiang province. These data indicate that *tmexCD1-toprJ1*-positive TNSKP have been persistently present in human.

It should be noted that *in vitro* transfer of *tmexCD1-toprJ1* only led to moderate tigecycline MIC increase by 8–16-fold, which was lower compared to that in *tet*(X) (64–128-fold). However, due to the efflux pump nature of *tmexCD1-toprJ1*, it not only mediates tigecycline resistance, but also may mediate resistance to other antimicrobials, such as ciprofloxacin, tetracycline and chloramphenicol.

It was surprising to find the high prevalence of *tmexCD1-toprJ1* in TNSKPs (52·4%) from the animal origins. It is likely that the *tmexCD1-toprJ1* had already disseminated in animals in China. Given the sharp differences in the prevalence of *tmexCD1-toprJ1-*positive TNSKP between animal origin and human origin (52·4% *vs.* 2·5%) as well as the phylogenetic findings, we suspect that *tmexCD1-toprJ1*-mediated tigecycline resistance originated in chickens and subsequently spread to human through plasmid-mediated gene transfer. Interestingly, tigecycline has never been introduced to veterinary practice, but other tetracyclines, such as oxytetracycline and doxycycline were heavily used in chickens [24]. It is likely that continuous abuse of those tetracyclines impose continuous selective pressure to facilitate the spread of *tmexCD1-toprJ1* gene in animal-derived isolates. Thus, it is of paramount important for multi-center surveillance and molecular epidemiological studies on the distribution and dissemination of *tmexCD1-toprJ1* in both veterinary side and clinical side.

The co-existence of *tmexCD1-toprJ1* with *mcr-8* in the same plasmid deserves much attention, as our conjugation assay and phylogenic analysis reveal a high transferability of the *tmexCD1-toprJ1*-*mcr-8* co-harbouring plasmid. Given this, further acquisition of carbapenem resistance genes by strains with *tmexCD1-toprJ1*-*mcr-8* co-harbouring plasmid, or vice versa, would inevitably lead to pan-drug-resistant strains. In fact, we found that the *tmexCD1-toprJ1*-*mcr-8* co-harbouring plasmid was indeed co-existed with *bla*_NDM_-harbouring plasmid in two strains. It is notable that one of the major *tmexCD1-toprJ1*-*mcr-8* co-harbouring plasmid types is IncFII plasmid. Previous studies from our groups and others have demonstrated that the major carbapenem resistance genes *bla*_KPC_-harbouring plasmid type is also IncFII plasmid [25]. Given the high prevalence of *bla*_KPC_-CRKP in China, it is highly likely that *tmexCD1-toprJ1*-*mcr-8* co-harbouring plasmid acquires *bla*_KPC_, making a “super-drug resistant” plasmid.

A new *mcr-8* variant *mcr-8·5* was identified in two isolates. *mcr-8* was recently reported in a CRPK from the pig in China [26]. Subsequently, *mcr-8* variants *mcr-8·2* and *mcr-8·3* were characterized in *K. pneumoniae* and *mcr-8·4* were found in *Raoultella ornithinolytica* [27, 28]. *mcr-1* was the first identified plasmid-mediated colistin-resistant gene, has gained extensive attention [11]. Interestingly, in the case of co-existence with *tmexCD1-toprJ1*, only *mcr-8* and its variant *mcr-8·5*, as well as *mcr-3·11* were identified*.* Of note, *mcr-1* and *mcr-8* display distinct preference to plasmids (i.e. *mcr-1* prefers IncI2 and IncX4, while *mcr-8* prefers IncFI and IncFII), while *mcr-8* displays similar plasmid preference to *tmexCD1-toprJ1* (i.e. both *mcr-8* and *tmexCD1-toprJ1* prefer IncFI and IncFII), it is likely that the common preference to the same plasmid determines the co-existence of *mcr-8* with *tmexCD1-toprJ1*.

Similar to *tmexCD1-toprJ1,* phylogenetic analysis also reveals that *mcr-8·5* was transmitted from animal to human. Colistin had been used as an animal feed additive in China for a long time, leading to the emergence, expansion and widespread of *mcr*-bearing plasmids. Fortunately, the Chinese Ministry of Agriculture announced a ban on use of colistin in animals in 2017 [29]. With the introduction of polymyxin B (Approved in Jan. 2018) and colistin (Approved in Jul. 2019) into clinical practice, active surveillance of those *mcr*-bearing plasmids is of great importance.

In summary, our study deserves much more global attention, as our data clearly point out that *tmexCD1-toprJ1*-mediated tigecycline-resistance mechanism is already present in isolates from patients. Even worse, we show that *tmexCD1-toprJ1*-*mcr-8* co-harbouring plasmid co-existed with *bla*_NDM_-harbouring plasmid in a single isolate, which is a truly “super-drug resistant bug,” already caused infections in patients. Thus, more effort is needed to elucidate the risk factors and clinical outcomes of patients with this “superbug.” Given that both *tmexCD1-toprJ1* and *mcr-8·5* are more likely to originate from animal, our data also emphasizes the urgent need for a “one-health” strategy and the global epidemiological surveillance landscape for the *tmexCD1-toprJ1-*harbouring plasmid, especially the *tmexCD1-toprJ1*-*mcr-8* co-harbouring plasmid.

## Supplementary Material

Supplemental Material
