# Supplementary material for: Co-existence of a novel plasmid-mediated efflux pump with colistin resistance gene mcr in one plasmid confers transferable multidrug resistance in Klebsiella pneumoniae
Source: Emerg Microbes Infect. 2020 May 31;9(1):1102–13. doi: 10.1080/22221751.2020.1768805 (PMC8284978; doi:10.1080/22221751.2020.1768805)
Supplement: Supplemental Material [file TEMI_A_1768805_SM3430.docx]

**Table S1. List of primers used in this study.**

| **Primer** | **Product** | **Sequence (5’–3’)** | **Purpose** | **Product length (bp)** | **Annealing temperature (◦C)** |
| --- | --- | --- | --- | --- | --- |
| *mcr-8*-F | *mcr-8* | CACTTTGGCAAACACTATGG | Presence verification and SNPs comparison | 1572 | 56 |
| *mcr-8*-R |  | TTTCTCTCTTACAACTGCGG |  |  |  |
| *mex*-F | *tmexCD1-toprJ1* efflux pump | TGAGTGATGAAGACCTTGGG | Cloning genes and constructing vectors | 7205 | 56 |
| *mex*-R |  | CACTCCTCTTGACCACCAAT |  |  |  |

**Table S2. MIC (μg/mL) of tetracycline of the transformants.**

| **Bacteria** | **Description** | **MIC (μg/mL) of tetracycline** |
| --- | --- | --- |
| *E.coli* DH5α | Recipient for transformantion | 1 |
| DH5α+pUC19 | Transformants | 1 |
| DH5α+pUC19-*tmexCD1* | Transformants | 8 |
| *K. pneumoniae* ATCC13883 | Recipient for transformantion | 2 |
| ATCC13883+pUC19 | Transformants | 2 |
| ATCC13883+pUC19-*tmexCD1* | Transformants | 8 |

**Table S3. Prevalence of *tmexCD1-toprJ1* efflux pump in *K pneumoniae* as determined by retrospective screening**

| **Programme name** | **Origin** | **Year** | **Total no. of isolates** | **No. of *K pneumoniae*** | **No. of tigecycline-non-susceptible *K pneumoniae*** | ***tmexCD1-toprJ1* positive isolates (%) / tigecycline-non-susceptible *K pneumoniae*** |
| --- | --- | --- | --- | --- | --- | --- |
| Animal 2013-2016 | Animal | 2013-2016 | 624 | 125 | 21 | 11 (52%)/21* |
| CRE network | Humans | 2012-2018 | 3355 | 2064 | 155 | 1 (0·6%)/155* |
| CMSS network | Humans | 2010 | 1258 | 170 | 23 | 0 /23 |
| CMSS network | Humans | 2012 | 1247 | 187 | 17 | 4 (23·5%)/17 |
| CMSS network | Humans | 2014 | 1437 | 199 | 16 | 0/16 |
| CMSS network | Humans | 2016 | 1401 | 205 | 19 | 0/19 |
| CMSS network | Humans | 2018 | 1214 | 222 | 6 | 1 (16·7%)/6 |

*All the *tmexCD1-toprJ1* positive isolates in the CRE network, and ten *tmexCD1-toprJ1* positive isolates in the Animal 2013-2016 have been subjected to whole genome sequencing and are listed in Table 1.

Table S4. Minimum inhibitory concentrations of tested antimicrobial agents for the bacterial that carry ***tmexCD1-toprJ1*** efflux pump.

| Isolates No. | MIC (µg/mL) | | | | | | | | | | | | | | | | | |
| --- | --- | --- | --- | --- | --- | --- | --- | --- | --- | --- | --- | --- | --- | --- | --- | --- | --- | --- |
|  | COL | TGC | MEM | IPM | ETP | MIN | CRO | FEP | FOX | CTX | CTC | CAZ | CSL | TZP | AMK | CIP | LVX |  |
| KA1 | 8 | 16 | 0.125 | 0.25 | 0.25 | 256 | 256 | 32 | 32 | 256 | 128 | 32 | 64 | >256 | >256 | >256 | 256 |  |
| KA2 | 8 | 1 | 0.125 | 0.25 | 16 | 64 | >256 | 128 | 16 | >256 | >256 | >256 | 128 | >256 | >256 | >256 | 128 |  |
| KA3 | 4 | 8 | 0.064 | 0.25 | 0.25 | 256 | 256 | 64 | 32 | 256 | 32 | 64 | 32 | >256 | >256 | >256 | 128 |  |
| KA4 | 2 | 8 | 0.125 | 0.25 | 0.25 | 128 | >256 | 32 | 32 | 256 | 64 | 32 | 32 | >256 | >256 | >256 | 256 |  |
| KA5 | 4 | 1 | 0.064 | 0.25 | 0.25 | 128 | >256 | 128 | 32 | >256 | 256 | 128 | 64 | >256 | >256 | >256 | 256 |  |
| KA6 | 16 | 16 | 0.032 | 0.125 | 0.25 | 32 | 2 | 1 | 256 | 4 | 8 | 16 | 2 | 16 | >256 | >64 | >64 |  |
| KA7 | 32 | 32 | 0.032 | 0.125 | 0.125 | 32 | 128 | 8 | 16 | 64 | 0.125 | 8 | 32 | 8 | >256 | >64 | >64 |  |
| KA8 | 0.125 | 32 | 0.032 | 0.125 | 0.5 | 32 | 64 | 2 | 256 | 16 | 4 | 4 | 16 | 16 | >256 | >64 | >64 |  |
| KA9 | 64 | 16 | 8 | 4 | 16 | 64 | >256 | 64 | >256 | >256 | 256 | >256 | >256 | >256 | >256 | >64 | >64 |  |
| KA10 | 32 | 16 | 8 | 8 | 32 | 64 | >256 | 64 | >256 | >256 | 256 | >256 | >256 | >256 | >256 | >64 | >64 |  |
| KA11 | 256 | 8 | 8 | 32 | 32 | 128 | >256 | 64 | >256 | 256 | 256 | >256 | >256 | >256 | >256 | >64 | >64 |  |
| KA12 | 256 | 8 | 8 | 4 | 32 | 64 | >256 | 64 | >256 | 256 | 256 | >256 | >256 | >256 | >256 | >64 | >64 |  |
| KA13 | 256 | 4 | 0.032 | 0.125 | 0.5 | 64 | 64 | 16 | 256 | 32 | 8 | 16 | 32 | 8 | >256 | >64 | >64 |  |
| KH1 | 32 | 16 | 0.032 | 0.125 | <=0.016 | 32 | 0.064 | 2 | 8 | 0.125 | 0.125 | 1 | 1 | 4 | 1 | 32 | 16 |  |
| KH2 | 64 | 16 | 0.032 | 0.125 | <=0.016 | 16 | 0.064 | 2 | 8 | 0.125 | 0.125 | 1 | 1 | 4 | 1 | 32 | 16 |  |
| KH3 | 16 | 8 | 4 | 4 | 32 | 64 | >256 | 64 | >256 | >256 | 128 | >256 | 256 | 256 | >256 | >64 | >64 |  |
| KA15 | 32 | 0.5 | 0.125 | 0.5 | 0.25 | 4 | >256 | 64 | 256 | >256 | 32 | >256 | 64 | >256 | >256 | 128 | 64 |  |
| KA16 | 32 | 0.5 | 0.064 | 0.5 | 0.25 | 4 | 16 | 2 | >256 | 8 | 4 | 256 | 4 | 128 | >256 | 128 | 64 |  |
| KA17 | 32 | 1 | 4 | >32 | >32 | 4 | >256 | 128 | >256 | >256 | >256 | >256 | 128 | >256 | >256 | 128 | 128 |  |
| KA18 | 32 | 0.5 | 4 | >32 | >32 | 4 | >256 | 128 | >256 | >256 | >256 | 256 | 64 | >256 | >256 | 256 | 128 |  |
| KA19 | 32 | 1 | 0.064 | 0.5 | 0.25 | 16 | 256 | 32 | 8 | >256 | 64 | 128 | 16 | >256 | 2 | 32 | 16 |  |
| KA20 | 32 | 0.5 | 0.125 | 0.5 | 1 | 4 | >256 | 64 | 256 | >256 | 128 | >256 | 64 | >256 | >256 | 128 | 64 |  |
| KH4 | 16 | 2 | 8 | 8 | 32 | 64 | >256 | 64 | >256 | >256 | >256 | >256 | 256 | >256 | 1 | 1 | 2 |  |
| KH5 | 16 | 4 | 4 | 16 | >32 | 16 | 256 | 32 | >256 | 128 | 64 | 32 | >256 | >256 | >256 | >64 | >64 |  |
| KH6 | 8 | 2 | 8 | 4 | 4 | 32 | >256 | >256 | 64 | >256 | >256 | >256 | 256 | >256 | 16 | >64 | >64 |  |
| KH7 | 8 | 1 | 0.032 | 0.125 | <=0.016 | 32 | 32 | 64 | 16 | 128 | 0.125 | >64 | 8 | 8 | >256 | >64 | >64 |  |
| KA14 | 4 | 1 | 0.032 | 0.125 | 0.5 | 32 | 32 | 1 | 256 | 64 | 128 | >256 | 32 | >256 | >256 | >64 | >64 |  |

*Antimicrobial agents are abbreviated as follows: COL, colistin; TGC, tigecycline; MEM, meropenem; IPM, imipenem; ETP, ertapenem; MIN, minocycline; CRO, ceftriaxone; FEP, cefepime; FOX, cefoxitin; CTX, cefotaxime; CTC, cefotaxime/Clavulanate; CAZ, ceftazidime; CSL, cefpoerazone-sulbactam; TZP, piperacillin-tazobactam; AMK, amikacin; CIP, ciprofloxacin; LVX, levofloxacin.*


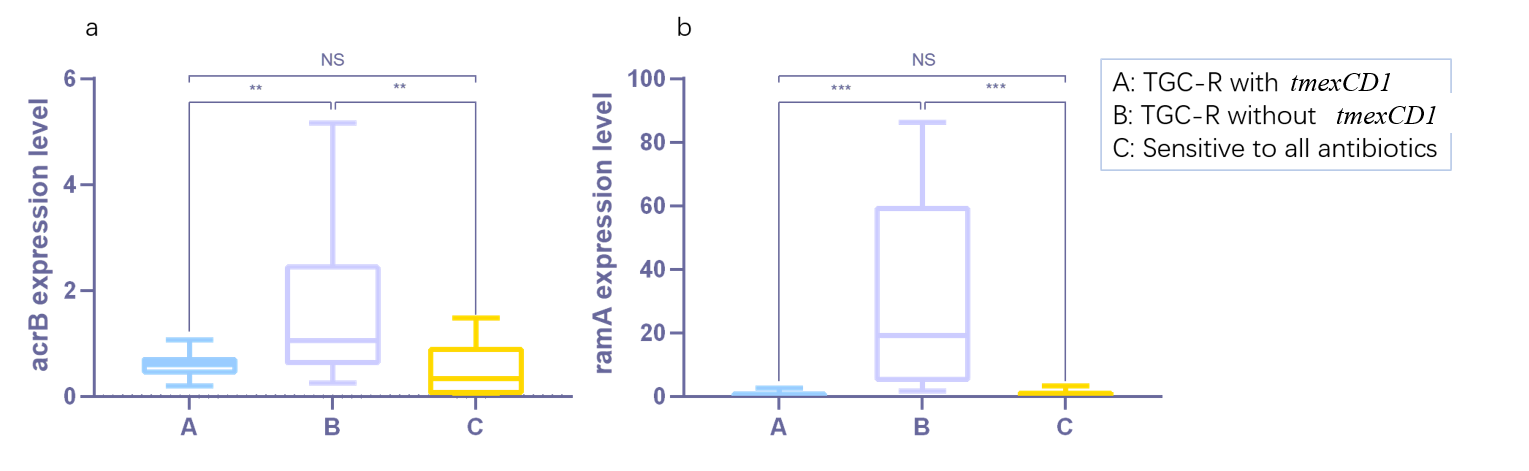


**Figue S1. The expression level of *acrB* and *ramA* in different group *K pneumoniae*. Group A carries a plasmid with *tmexCD1* and is resistant to tigecycline; Group B is resistant to tigecycline without *tmexCD1*; Group C is Sensitive to almost all antibiotics**

**Figue S2. The effect of carrying plasmid with *tmexCD1*–*toprJ1* efflux pump on growth. The green line represents the *E.coli* J53, and the yellow line represents the transconjugants that carrying the plasmid of *mcr-8*, while the red line represents the transconjugants that carrying the plasmid of *tmexCD1* and *mcr-8***
